# Clinical implications of chromatin accessibility in human cancers

**DOI:** 10.18632/oncotarget.27584

**Published:** 2020-05-05

**Authors:** Yuexin Liu

**Affiliations:** ^1^ Department of Bioinformatics and Computational Biology, The University of Texas MD Anderson Cancer Center, Houston, Texas, USA

**Keywords:** ATAC-seq, chromatin accessibility, TCGA, promoter, survival

## Abstract

Assay for transposase-accessible chromatin using sequencing (ATAC-seq) has not yet been widely used in cancer research. Clinical implications of chromatin accessibility assessed by ATAC-seq profiling in human cancers especially in a large patient cohort is largely unknown. In this study, we analyzed ATAC-seq data in 404 cancer patients from the Cancer Genome Atlas, representing the largest cancer patient cohort with ATAC-seq data, and correlated chromatin accessibility with patient demographics, tumor histology, molecular subtypes, and survival. Our results showed that chromatin accessibility varies from chromosome to chromosome, and is different in different genomic regions along the same chromosome. Chromatin accessibility especially on the X chromosome is strongly dependent on patient sex, but not much on patient age or tumor stage. Striking difference in chromatin accessibility is observed between lung adenocarcinoma and lung squamous cell carcinoma, the two most common histological subgroups in lung cancer. Furthermore, chromatin accessibility was different between basal and non-basal breast cancer. Finally, we identified prognostic peaks in the promoter regions that were significantly correlated with survival. In particular, we identified six peaks in the ESR1 gene promoter region in the ATAC-seq profiling and found that the peak about 247 bp away from the transcription start site was significantly associated with better survival. In conclusion, our study provides an alternative mechanism underlying tumor prognosis.

## INTRODUCTION

Cancer is a heterogeneous disease with a diversity of cell types which thus play a deterministic role on patient outcome or therapeutic responses. The chromatin structure and compaction play an essential role in genome accessibility and are an essential determinant of cellular phenotypes [1]. Chromatin accessibility is a hallmark of transcription factor binding and such DNA regulatory elements as promoters and enhancers [2]. The assay for transposase-accessible chromatin using sequencing (ATAC-seq) employs hyperactive Tn5 transposase for highly efficient cutting of exposed DNA and simultaneous ligation of adapters which are then subject to next-generation sequencing. Therefore, ATAC-seq has enabled the genome-wide profiling of chromatin accessibility in primary human cancers. Different from Dnase I hypersensitive sites sequencing (DNase-seq), ATAC-seq requires smaller quantities of frozen tissue (~50,000 cells) [3]. Low requirements on the amount of input material makes it possible to assay rare but important cellular subtype [4] or to combine with the currently emerging single-cell sequencing technique [5–7]. In addition, the protocol for performing ATAC-seq profiling is simple and requires only a few hours in total [8]. Due to these unique characteristics along with improved protocols [9], ATAC-seq has emerged as a widely-adopted technique for global chromatin accessibility analysis, and has received intensive research attention lately [10–12]. Unexpectedly, there is little research on cancer study by using ATAC-seq technique with a limited number of cancer types such as prostate cancer, pancreatic cancer and hematological malignancy [13–15].

Recently, the Cancer Genome Atlas (TCGA) performed ATAC-seq on 410 tumor samples derived from 404 unique donors and generated a catalog of chromatin accessibility in human cancers [16]. The samples covered 23 different cancer types which were representative of the diversity of human cancers. Although the TCGA effort resulted in a novel and unique dataset in a relatively large patient cohort, the clinical implications of chromatin accessibility in human cancers have not been systematically investigated yet in the original TCGA publication [16], and remain largely unknown.

In this study, we will leverage the full scope of the TCGA ATAC-seq data to interrogate the chromosomal landscapes of regulatory elements in the human genome. Different from the TCGA work on enhancers [16], the present study is primarily focusing on regulatory peaks particularly in the promoter regions. We will further integrate ATAC-seq data along with the patient clinical annotations or molecular characteristics to determine the association between chromatin accessibility in the promoter regions and patient demographics such as sex, age, tumor stage and histology, molecular subtype, and patient survival.

## RESULTS

### Chromosomal landscape of chromatin accessibility in human cancers

**Figure 1:**
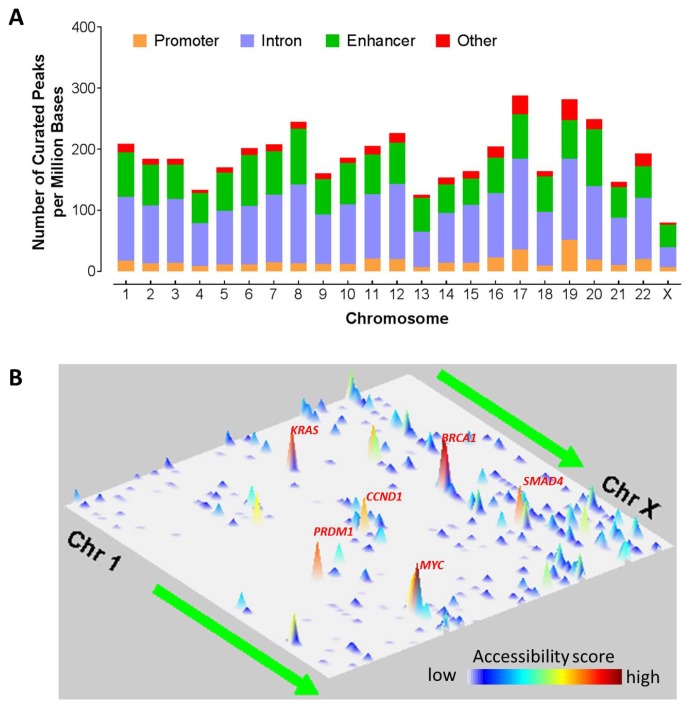
Chromosomal landscape of chromatic accessibility in human cancers. (**A**) Distribution of all the regulatory elements such as promoter, enhancer, intron, and other elements across chromosomes. Here the other elements denote elements located at the exonic region, or 3′ UTR, or 5′ UTR. Color indicates the type of genomic region overlapped by the peak. UTR, untranslated region. (**B**) Genome landscapes of chromatin accessibility. The chromatin accessibility scores indicate the likelihood of chromatin openness and are plotted in two-dimensional space representing chromosomal positions of human genome assembly (GRCh38). One dimension consists of the 23 chromosomes from Chr1 to ChrX and the other dimension indicates the genomic coordinates on a chromosome from p arm to q arm. Correlation of the colors and accessibility scores is indicated by the accompanied colorbar.

ATAC-seq fastq files were first processed using the PEPATAC pipelines and hg38 genome build to produce aligned, de-duplicated BAM files. Peaks with a fixed width of 501 bp were then called by using the MACS2 method and further curated via an iterative removal process and normalization method [16], resulting in a set of high-quality fixed-width peaks that can be used in downstream analyses. To assess the chromosomal landscape of chromatin accessibility, we first calculated the number of curated peaks per megabase (PPMB) in the human genome for each of the 23 chromosomes (Chr 1, Chr 2, …, Chr X) (Figure 1A). The median density is about 186 PPMB and the peak densities vary from chromosome to chromosome. Chr17 and Chr19 had the largest number of peaks per megabase (~280 PPMB), suggesting that these two chromosomes are frequently accessible. Following that are chromosomes 20 and 8 that had about 220 PPMB. On the other hand, Chr X has the least peak density of around 80 PPMB, likely due to the inactivation of X chromosome via DNA methylation [17]. This is consistent with the hypothesis that methylated DNA is less accessible [18]. In addition, Chr 4 and Chr 13 also had relatively low peak density, suggesting that these two chromosomes were less likely accessible. For each of the four peak categories (i. e., promoter, enhancer, intron, and others), the variation is also different. For instance, Chr 19 has the highest promoter peak density of over 50 promoters per million bases. On the other hand, Chr 8 and Chr 20 have the highest enhancer density of over 90 enhancers per million bases.

In addition, the peaks are not uniformly distributed but vary with genomic regions on the chromosomes (Figure 1B). Given that the majority of the cancer types in this cohort had a small number of patients, we analyzed all the cancer types together for this analysis. Two consecutive cytoband regions (q21.31 and q21.32) on Chromosome 17 are the most likely accessible region with the highest accessibility score. Interestingly, the region contains *BRCA1* gene and homeobox B family genes. Chromosome 18 at q21.32-q33 is easily accessible and contains genes such as *SMAD4*, and *BCL2* etc. In addition, Chr12p12.1 (containing genes such as *KRAS* and *SOX5*), Chr8q24.21 (containing *MYC* gene), and Chr11q13.3 (containing *CCND1* gene) also has high accessibility. Chr6q21 has a high accessibility score and contain immune master regulators (*PRDM1* and *ROS1*). Chromatin structure alteration in promoter regions due to DNA methylation are known to intimately associated with gene expression regulation [19]. As a result, we will focus on ATAC-seq analysis on chromatin accessibility in the promoter regions, different from the original TCGA work on enhancers [16].

### Association of chromatin accessibility with patient demographics

**Figure 2:**
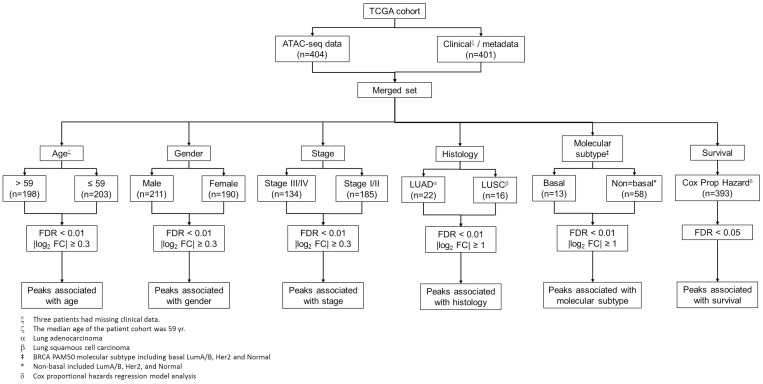
Identification of clinically-associated peaks in the promoter regions. Analysis flow chart for identifying peak elements in the promoter regions which are associated with clinicopathologic characteristics, histological and molecular subtypes, and survival.

**Figure 3:**
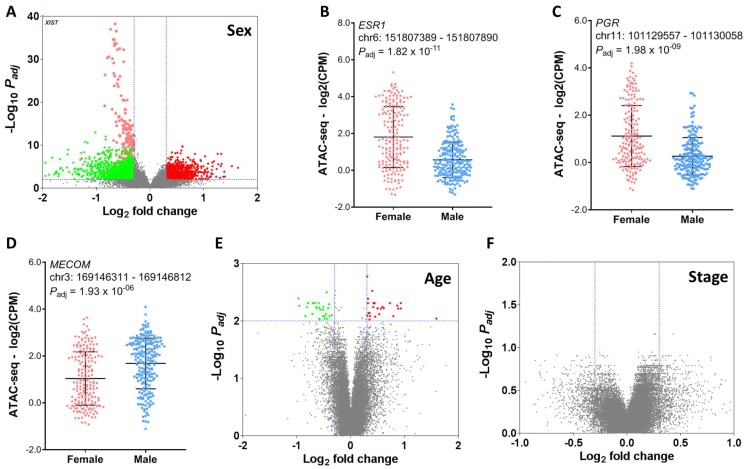
Association of chromatin accessibility with patient demographics. (**A**) Difference in chromatin accessibility between male and female patients. Dots in red indicate ATAC-seq peaks that have significantly intensity in the male patients. Dots in green indicate ATAC-seq peaks that have significantly higher intensity peaks in the female patients. Peaks within the (**B**) *ESR1* and (**C**) *PGR* gene promoter region are significantly higher in the female patients. (**D**) A peak within the *MECOM* gene promoter region is significantly higher in the male patients. (**E**) Difference in chromatin accessibility between young and old patients. (**F**) Difference in chromatin accessibility between advanced-stage (III or IV) and early-stage (I or II).

To obtain a comprehensive view of clinical implications of chromatin accessibility, we seek to identify peak elements in the promoter regions which are significantly associated with clinicopathologic characteristics such as age, sex, and stage, histological and molecular subtypes, and patient survival (Figure 2). We next aimed to characterize the effect of patient demographics such as age at diagnosis, sex and tumor stage on chromatin accessibility in human cancer (see Methods). We first compared peak counts in the promoter region between male and female patients in the TCGA cohort. Total 2534 peaks exhibited significant differences in chromatin accessibility (FDR < 0.01 and |log2 FC| > 0.3), among which 1035 peaks were higher in male patients and 1499 peaks were higher in female patients. Interestingly, the peaks with the most statistical significance were located on chromosome X and were significantly higher in the female patients. One of these peaks was in the promoter region of the non-coding RNA (*XIST*) (Figure 3A). Openness of the chromatin in the proximity of *XIST* may cause upregulation of *XIST* RNA, which subsequently triggers X-chromosome inactivation [20–22]. In addition to peaks on the X chromosome, the promoter peaks near the hormone receptors such as estrogen receptor alpha (encoded by *ESR1* gene) and progesterone receptor (encoded by *PGR* gene) also exhibited significantly higher intensity in female patients (Figure 3B and 3C). Conversely, an ATAC-seq peak in the *MECOM* gene promoter region is significantly higher in male patients (Figure 3D). The regulatory elements near the *MECOM* gene were recently reported to be associated with a subgroup in KIRP [16]. With the same criteria, we detected only 56 peaks showing significant differences in chromatin accessibility between young and old patients (Figure 3E). Furthermore, we didn’t detect any regions differentially accessible between advanced-stage and early-stage (Figure 3F), suggesting that tumor aggressiveness has little impact on compaction of regulatory elements. This is consistent with a previous report that very few peaks were differentially observed between cases and controls [23]. A study reported a small number of chromatin accessibility differences between two CLL subtypes with striking difference in prognosis [14]. In all, our results indicate that chromatin accessibility is strongly dependent upon sex, but not upon age and stage.

### Association of chromatin accessibility with histological and molecular subtypes

**Figure 4:**
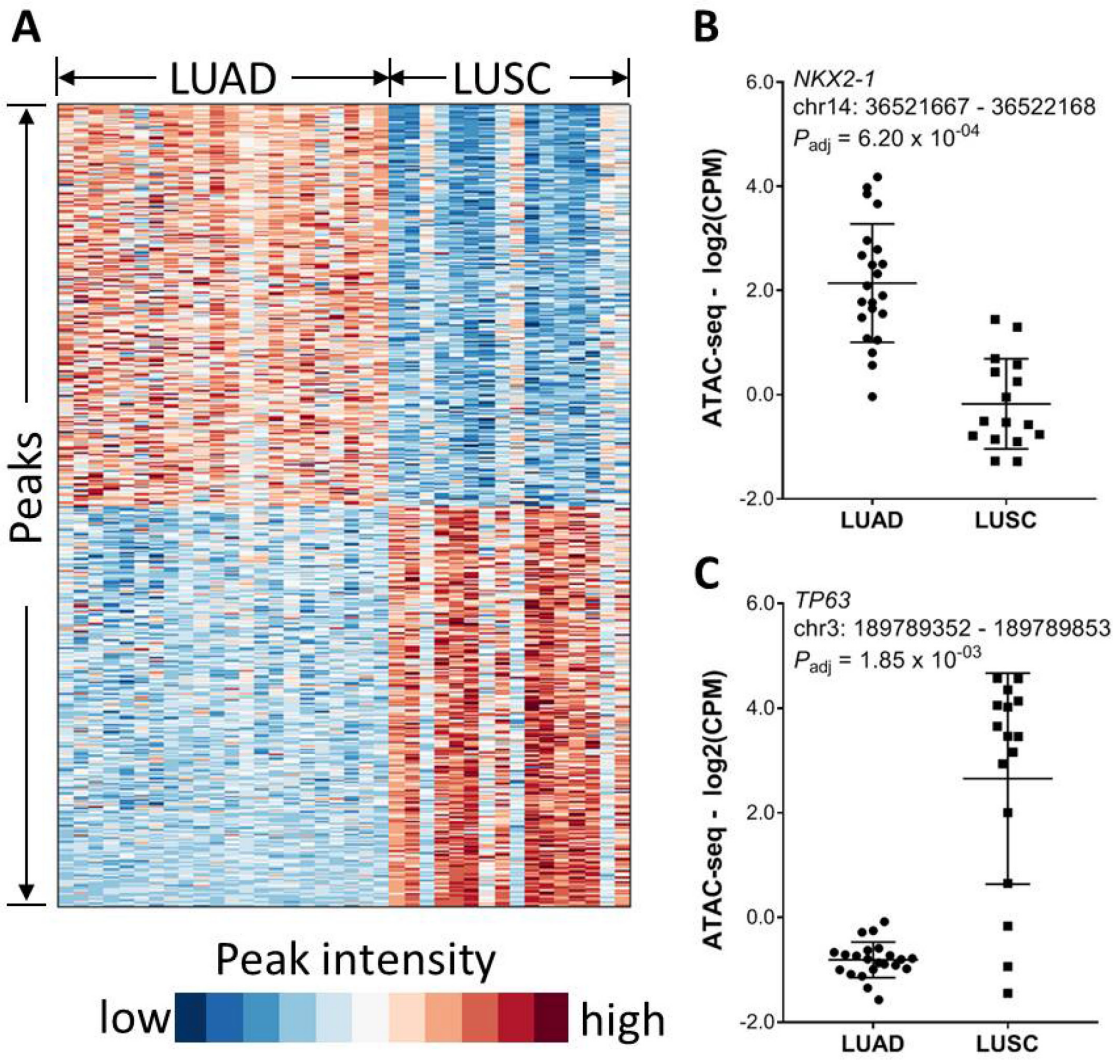
Association of chromatin accessibility with tumor histology. (**A**) Significantly differential chromatin between LUAD and LUSC (FDR < 0.01 and magnitude in fold change ≥ 2). (**B**) An ATAC-seq peak within the *NKX2-1* gene promoter region is significantly higher in LUAD. (**C**) An ATAC-seq peak within the *TP63* gene promoter region is significantly higher in LUSC.

We next investigate the effect of tumor histology on chromatin accessibility. Lung adenocarcinoma (LUAD) and lung squamous cell carcinoma (LUSC) are two most common histological subtypes in lung cancer. While comparing chromatin accessibility between LUAD and LUSC, we identified total 1723 differential chromatin elements (FDR < 0.01 and fold change ≥ 2), among which 858 peaks were significantly higher in the LUSC cases while 865 peaks were higher in the LUAD cases (Figure 4A). A peak located within the *NKX2-1* promoter region and about 769 bp away from the transcription start site (TSS) had about 6- fold higher intensity in the LUAD cases (Figure 4B). On the other hand, a peak located within the *TP63* promoter region and about 57 bp away from the TSS had over 19- fold higher intensity in the LUSC cases (Figure 4C). Consistently, it was recently reported that *NKX2-1* has a stronger footprinting and flanking signal in LUAD than in LUSC in contrast to a stronger signal that was observed for *TP63* in LUSC than in LUAD [16].

**Figure 5:**
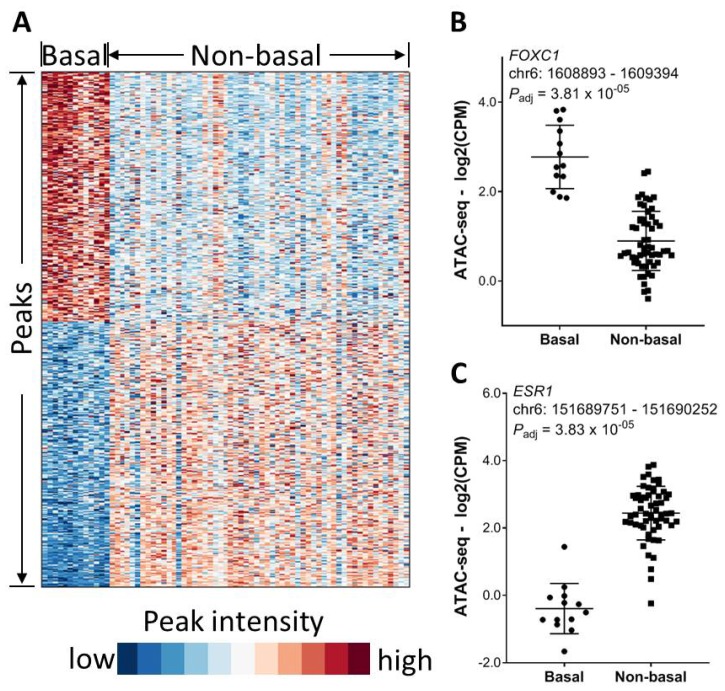
Association of chromatin accessibility with molecular subtype. (**A**) Significantly differential chromatin between basal and non-basal BRCA patients (FDR < 0.01 and magnitude in fold change ≥ 2). (**B**) An ATAC-seq peak within the *FOXC1* gene promoter region is significantly higher in basal BRCA patients. (**C**) An ATAC-seq peak within the *ESR1* gene promoter region is significantly higher in non-basal BRCA patients.

In addition to tumor histology, we next investigated the impact of molecular subtype on chromatin accessibility. We took breast cancer as an example because it had the largest number of samples in this ATAC-seq patient cohort. In a similar manner, we identified total 2590 differential chromatin elements between basal and non-basal BRCA patients (FDR < 0.01 and fold change ≥ 2), among which 1334 peaks were significantly higher in the non-basal cases while 1256 peaks were higher in the basal cases (Figure 5A). A peak about 828 bp away from the *FOXC1* TSS had significantly higher intensity in the basal cases (Figure 5B). The recent paper [16] showed that *FOXC1* is a transcription factor specific to BRCA basal group. As expected, a peak about 494 bp away from the *ESR1* TSS site had significantly higher intensity in the non-basal BRCA cases as compared to the basal BRCA cases (Figure 5C).

### Association of chromatin accessibility with survival

**Figure 6:**
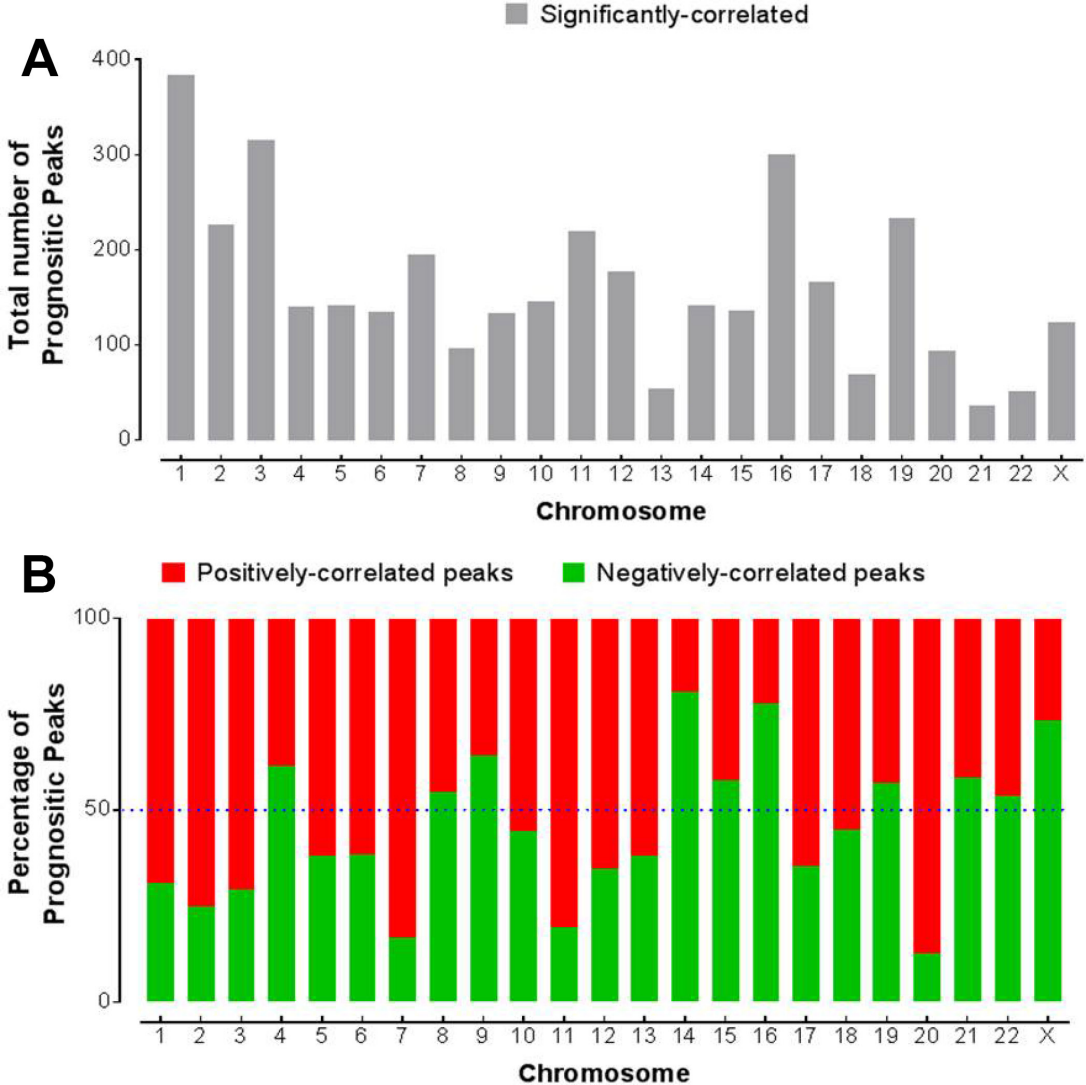
Association of chromatin accessibility with survival. (**A**) Distribution of the peak elements within the promoter region that are significantly correlated with overall survival (*P_adj_* < 0.05). (**B**) The percentage of those positively- or negatively- correlated peaks among the prognostic peaks in each chromosome.

To understand the relationship of chromatin accessibility with clinical outcome, we next correlated the peak intensity with patient survival. Given that most of the cancer types had a small number of patients, we analyzed all the cancer types together for estimating the survival. We first applied the Cox proportional hazards regression model to correlate the peak signal in the promoter regions with overall survival. Total 3724 peaks were found to be significantly correlated with survival (Cox *P* < 0.05 after multiple testing correction). These peaks were unevenly distributed across chromosomes. Chrs 1, 3 and 16 had the largest number (> 300) of prognostic peaks while Chrs 21, 22 and 13 have the least (< 60) number of prognostic peaks (Figure 6A). Not only the total number of prognostic peaks, but also the percentage of either positively or negatively-correlated peaks varied with chromosomes (Figure 6B). The majority of the prognostic peaks on Chrs 1, 2, 3, 7 and 20 were positively correlated with survival, meaning higher intensities of these peaks were correlated with worse survival. On the other hand, the majority of the prognostic peaks on chromosomes 14, 16 and X were negatively correlated with survival, meaning higher intensities of these peaks were correlated with better survival.

**Figure 7:**
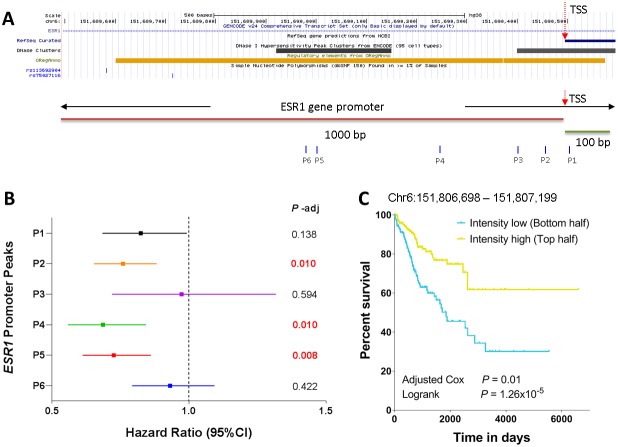
Correlation of *ESR1* promoter peaks with survival. (**A**) *ESR1* gene promoter region covering −1000 bp and +100 bp from the transcription start site. The DNase hypersensitivity clusters, transcription factor binding regions, and common SNP sites are also indicated. The six ATAC-seq peaks within the *ESR1* promoter region are also indicated. (**B**) Correlation of the 6 ATAC-seq peaks with patient survival via Cox proportional hazard model analysis. The BH-adjusted *P* values are also indicated. (**C**) Kaplan-Meier plot of the peak intensities located at P4. The patients were categorized into two groups based on the median signal.

It is demonstrated above that the peaks in the *ESR1* promoter region are significantly associated with female patients and BRCA non-basal subtype. We next seek to investigate whether or not these peaks are correlated with patient survival. Figure 7A shows the *ESR1* gene promoter region defined as occurring between –1000 bp and +100 bp of the TSS of the *ESR1* gene. Region shown in this plot represents chromosome 6 (chr6): 151689496 to 151690596. The *ESR1* promoter region has a broad transcription factor binding sites indicated by the orange track. The transcription factors that have binding sites in this region include forkhead box A1 (*FOXA1*), transcription factor AP-2 gamma (*TFAP2C*) and CCCTC-binding factor (*CTCF*) [24]. Two known GWAS single nucleotide polymorphisms rs113692904 and rs75027116 were also found in this region. Interestingly, the *ESR1* promoter also contains two DNase I hypersensitivity peak clusters (data were obtained from ENCODE). The first cluster is about 230 bp long, locating from chr6:151,689,921 – 151,690,150 (6q25.1). The second cluster is about 250 bp long, locating from chr6:151,690,401 – 151,690,650 (6q25.1). Total 6 ATAC-seq peaks were identified in the *ESR1* promoter region, which are −516 bp (referred to as P6: the sixth site in the promoter, and thereafter), −494 bp (P5), −247 bp (P4), −91bp (P3), −39 bp (P2), and 8 bp (P1), respectively, away from the TSS (Figure 7A). Five of these six ATAC-seq peaks are concordant with the DNase hypersensitivity clusters where P6 and P5 are in the first cluster and P1, P2, and P3 are in the second cluster. The high overlap of regulatory regions between the DNase clusters and ATAC-seq peaks demonstrates the robustness of the two platforms and the consistency of the results ever obtained. In addition to these concordant peaks, there is one peak (P4) which is identified by the ATAC-seq profiling but not contained in the DNase hypersensitivity clusters. Correlation of the intensities of these six peaks with survival showed that three ATAC-seq peaks (P2, P4, and P5) were significantly correlated with survival with hazard ratios of < 1, meaning that the higher intensity of these peaks is associated with better survival (Figure 7B). No significant correlation with survival was observed for the other three peaks. Especially the newly identified ATAC-seq peak (P4) was significantly correlated with survival (HR = 0.686, 95% Confidence Interval = 0.558 − 0.843, *P_adj_* = 0.01). We next divided the patients into two groups based on the intensity of this ATAC-seq peak (P4), patients with intensity higher than the median value were grouped into the “Intensity high” group and patients with intensity lower than the median value were grouped into the “Intensity low” group. The Kaplan-Meier analysis showed that patients in the high group had significantly better survival than those in the low group (Logrank *P* = 1.26 × 10^−5^, Figure 7C).

## DISCUSSION

In this study, we carried out systematic ATAC-seq data analysis in 404 TCGA patient samples spanning 23 different cancer types. Chromatin accessibility varies within and between chromosomes. We identified several fragile regions on the chromosomes that contained well-known oncogenes or tumor suppressor genes. We found that chromatin accessibility exhibited a striking difference between male and female patients, and not much dependent upon patient age and tumor stage. We also showed that chromatin accessibility strongly dependent upon tumor histology and molecular subtypes and further identified several sites that were specifically correlated with lung histology and BRCA subtypes. Finally, we identified prognostic peaks in the promoter region that were significantly correlated with survival and further demonstrated that these prognostic peaks varied from chromosome and chromosome. As an example, we showed that the peaks in the *ESR1* promoter region exhibited prognostic values.

By analyzing this unique and novel ATAC-seq data set, we uncovered several previously unappreciated aspects of chromatin accessibility in human cancers. To our best knowledge, this is the first time with such effort to investigate the chromosomal landscape of chromatin accessibility in a systematic manner. Chromatin accessibility is different not only from chromosome to chromosome, but also from region to region along the same chromosome. Interestingly, we identified several susceptibility loci that were most likely (assessed by accessibility score) accessible in the genome. The region with the highest accessibility score contained the *BRCA1* gene. It is known that germline mutation of *BRCA1* is a risk factor for ovarian and breast cancer [25] and BRCA1 carrier is sensitive to drugs that targeted the DNA damage and repair pathway [26]. The region with the second highest score contained the *MYC* gene. *MYC* is a well-known oncogene and amplified in a wide array of human cancers [27]. Other susceptible regions contain *CCND1, KRAS, SMAD4,* and *PRDM1* genes. Similar to *MYC, CCND1* is frequently amplified in the squamous cell carcinoma [28]. Instead of copy-number amplification, *KRAS* is frequently mutated in a wide array of tumors including lung cancer [29] and pancreatic cancer [30]. *SMAD4* is an important factor in the TGFβ pathway and also frequently altered at the genomic level [31] and *PRDM1* is recently reported to be the master regulator of pan-immune response [32].

The relationship between chromatin accessibility and aging has been previously reported in yeast and mammals [33]. Dependence of chromatin structure on aging is somehow cell-specific. Human CD8 T cells change chromatin accessibility with aging [34]. The differentiated cells such as filbroblasts has a wide variability of chromatin structure during aging, and on the other hand the human stem cell has a well-maintained chromatin structure during aging [35]. Similar to the stem cells, we found little change in chromatin structure in human tumor cell as a function of age. We understand that aging has important impact on cellular phenotypes, but the mechanism that aging has litter effect on chromatin accessibility in human tumor cells is elusive and deserves further investigation.

One limitation of our study is that the susceptibility regions are relatively broad and contain many other genes in addition to those oncogenes or tumor suppressor genes mentioned above. This should be taken into account while interpreting these data. Secondly, we analyzed all the cancer types together for the sake of statistical power consideration because some cancer types did not have enough samples. Tissue origin may play an important role in some of the analyses. Cancer-specific analysis may be required in the future when more samples are profiled via ATAC-seq. Because the ATAC-seq data from normal samples were not available in the current TCGA patient cohort, we did not systematically investigate the markers or regions that can be used for cancer detection. To further understand the causal effect of the findings identified in this study, functional validation is required.

In conclusion, chromatin accessibility has important clinical implications in human cancers and our results provide an additional perspective in tumor initiation and progression.

## MATERIALS AND METHODS

### Patient samples and ATAC-seq data

For the ATAC-seq data, we used the normalized count matrix obtained from TCGA [16], in which each row corresponds to a peak and the columns contain the sample IDs and the following data: the genomic region of the peak (including chromosome number, start and end coordinates), peak width (always 501 bp in this study), annotation (i. e., promoter/intron/distal/3′ UTR/5′ UTR/exon), the closest gene including Entrez gene ID, ENSEMBL gene name, and gene symbol, the distance to TSS. A prior count of five was first added to the raw count matrix from which we calculated the mean counts per million mapped reads (CPM), which we referred to as peak intensity in this study. The prior count was used to lower the contribution of variance from elements with lower count values. The resultant matrix was then log2 transformed, and then followed by a quantile normalization. We then averaged the values across all technical replicates and all biospecimens belonging to the same TCGA sample, resulting in total 404 unique TCGA samples, covering 23 different cancer types. Note that each cancer type was not represented by an equal number of samples (Table 1). The most common type was breast cancer with 74 patients while the least was the cervical cancer with only 2 patients. Promoter region is defined as within -1000 to 100bp to TSS sites. The promoter peak to gene mapping information is derived from if a peak summit locates within the promoter region of a gene. The clinicopathologic data of these patients as well as the survival data were obtained from the literatures [16, 36]. Note that there were three samples that had ATAC-seq data but did not have clinical data. Access to the TCGA ATAC-seq database was approved by the National Cancer Institute. MD Anderson Cancer Center waved the requirement for ethical approval of this analysis because of the registry contains only de-identified data. Written consent was obtained from all living patients.

**Table 1:** Distribution of patient samples in the TCGA ATAC-seq data set among the 23 cancer types that are sorted in descending order by their percentage in the cohort

Tumor type	Description	Patients (*N* = 404)	Percentage (%)
BRCA	Breast Invasive Carcinoma	74	18.3
COAD	Colon adenocarcinoma	38	9.4
KIRP	Kidney renal papillary cell carcinoma	34	8.4
PRAD	Prostate adenocarcinoma	26	6.4
LUAD	Lung adenocarcinoma	22	5.4
STAD	Stomach adenocarcinoma	21	5.2
ESCA	Esophageal carcinoma	18	4.5
LIHC	Liver hepatocellular carcinoma	17	4.2
KIRC	Kidney renal clear cell carcinoma	16	4.0
LUSC	Lung squamous cell carcinoma	16	4.0
THCA	Thyroid carcinoma	14	3.5
LGG	Brain lower grade glioma	13	3.2
SKCM	Skin cutaneous melanoma	13	3.2
UCEC	Uterine corpus endometrial carcinoma	13	3.2
BLCA	Bladder urothelial carcinoma	10	2.5
ACC	Adrenocortical carcinoma	9	2.2
GBM	Glioblastoma multiforme	9	2.2
HNSC	Head and neck squamous cell carcinoma	9	2.2
PCPG	Pheochromocytoma and paraganglioma	9	2.2
TGCT	Testicular germ cell tumors	9	2.2
MESO	Mesothelioma	7	1.7
CHOL	Cholangiocarcinoma	5	1.2
CESC	Cervical squamous cell carcinoma and endocervical adenocarcinoma	2	0.5

### Peak calling and curation

The methodology how to obtain and curate the peaks in the ATAC-seq data set has been described in details in the original TCGA publication [16]. ATAC-seq fastq profiles were first processed and converted into aligned, de-duplicated BAM files by using the PEPATAC pipeline (http://code.databio.org/PEPATAC/) and the hg38 genome build. Peak calling was performed for each sample using the MACS2 method with parameters “—shift -75 –extsize 150 –nomodel –call-summits –nolambda –keep-dup all –p 0.01” and then equally extended on both sides to a final width of 501 bp. The peaks were then subjected to multi-step curations. We used an iterative removal procedure to retain the most significant peaks by removing the overlapping ones, and then normalized peak calls for sample quality and total sequencing depth by dividing each individual peak score by the sum of the entire peak scores in the given sample divided by 1 million.

We next filtered out any peaks that were observed in only one sample, and any peaks that mapped to the Y chromosome or spanned a genomic region containing “N” nucleotides. Finally, we re-normalized the data for each cancer type and then re-used the iterative removal procedure to generate a high-quality, reproducible, and fixed-width “pan-cancer peak set” that could be used for cross-cancer comparisons.

### Chromosomal landscape

Starting with the normalized count matrix, we extracted the data for each of the following four categories such as enhancer, promoter, intron and others, where ‘others’ in this study indicated the peaks located at either exon or 3′ UTR or 5′ UTR. For each of the four categories, we then counted the number of peaks in each of the 23 chromosomes adjusted by the chromosomal length (Human genome assembly GRCh38), and then calculated the number of curated peaks per million bases.

Using a similar approach as described previously [37], we seek to construct the chromosomal landscape. In brief, we divide each of the 23 chromosomes (from 1 to X) into 92 evenly spaced segments and then count the total number of peaks in each of those segments. Since the lengths of different chromosomes are different, the length of the segment are different. Next we normalize the total number of peaks by the segment lengths (peak density). The peak density per segment can be used to characterize chromatin accessibility for that region. To further determine the statistical significance of the calculated chromatin accessibility, we randomly permuted the chromosome numbers and genomic locations in the original input data, and then calculated the peak densities for each of those segments in the same manner. This process was repeated 10^6^ times. For each of those segments, the peak densities of those random permutations generated a null distribution, from which we can calculate the nominal P value of the actual peak densities to this null distribution. The –log (*p* value) was used as a score to characterize the accessibility.

### Clinicopathological correlation

Given that some cancer types such as GBM, LGG, PCPG, PRAD, and UCEC did not have the stage information and some samples had missing stage data, overlapping with the ATAC-seq data resulted in total 319 samples that had both stage and ATAC-seq data including 134 advanced-stage (III or IV) and 185 early-stage (I or II) diseases, respectively. We then compared the signal intensities of the peaks within the promoter region between these two stage groups. The statistical significance was assessed via Mann-Whitney test adjusted by Benjamini-Hochberg (BH) multiple-testing, and peak difference in magnitude was calculated as log2 (fold change) where fold change is defined as peak values in the advanced-stage disease divided by peak values in the early-stage disease

To investigate the gender effect on the chromatin accessibility, we performed statistical analysis to compare peaks in the promoter region between male and female patients. Total 190 female patients and 211 male patients were included in this analysis. The statistical significance was assessed via Mann-Whitney test adjusted by BH multiple-testing, and peak difference in magnitude was calculated as log2 (fold change) where fold change is defined as peak values in the male patients divided by peak values in the female patients.

To investigate the age effect on chromatin accessibility, we first calculated the median age of the patient cohort (which was 59 years old in this case) and then split patients into two groups based on the patient age. Patients with age less than or equal to 59 were grouped into an age-low group (*n* = 203), and patients with age greater than 59 were grouped into an age-high group (*n* = 198). Again, we then compared the peaks between these two groups via Mann-Whitney test adjusted by BH multiple-testing and peak difference in magnitude was calculated as log2 (fold change) where fold change is defined as peak values in the age-high group divided by peak values in the age-low group.

### Correlation of chromatin accessibility with histological and molecular subtypes

We first aimed to identify differential peaks between LUAD and LUSC, the two common histological groups of lung cancer. In this cohort, we had 22 LUAD cases and 16 LUSC cases. The statistical significance in peak differences between these two histological groups was assessed via Mann-Whitney test adjusted by BH multiple-testing.

We next seek to correlate the peak intensity with BRCA Pam50 molecular subtypes. Among the 74 BRCA patients with ATAC-seq data, 3 patients did not have the Pam50 subtype information. Therefore, 71 BRCA cases in this cohort had both ATAC-seq data and Pam50 data, including 13 basal, 29 LumA, 16 LumB, 10 Her2, and 3 Normal. Compared to the other subtypes, basal BRCA patients had striking differences in clinical outcome and therapeutic response. Therefore in this study we compared the basal patients versus the non-basal BRCA patients (LumA/B, Her2, and Normal). The statistical significance in peak differences between these two molecular subtypes was assessed via Mann-Whitney test adjusted by BH multiple-testing. The heatmap showing the ATAC-seq peak signal differences between histological and molecular subtypes were generated by using the Matlab software, version 9.6 (MathWorks, Inc, Natick, MA).

### Survival analysis

After filtering out patients with survival data of equal to zero, we obtained 393 samples that had both ATAC-seq and survival data. Two different methods were utilized to examine the association of chromatin accessibility with patient survival. We first applied Cox proportional hazards regression models to correlate all the peaks in the promoter region with survival. In this analysis the peak intensity was treated as a continuous variable and Wald’s test was used to assess statistical significance. We also used the Kaplan-Meier survival analysis to compare the survival rate of the dichotomized groups. In brief, patients were dichotomized into either high-intensity or low-intensity group based on the median intensity for each peak, and the survival difference between these two groups was assessed with a log-rank test.

### Statistical analysis

We used the nonparametric Mann-Whitney test for comparison of peak intensities between two dichotic groups. The Kaplan-Meier method was used to evaluate survival difference between dichotic groups and statistical significance was assessed via log-rank test. All the p-values were adjusted by BH multiple testing unless otherwise specified. All statistical tests were two-sided, and a *P* value of less than 0.05 was considered significant. The calculations and graphs were made with Matlab, version 9.6 (MathWorks, Inc, Natick, MA), and GraphPad Prism, version 7.03 (GraphPad Software, Inc., La Jolla, CA).

## Abbreviations

ATAC-seq: assay for transposase-accessible chromatin using sequencing
TCGA: The Cancer Genome Atlas
LUAD: lung adenocarcinoma
LUSC: lung squamous cell carcinoma
BRCA: breast carcinoma
ESR1: estrogen receptor alpha
TSS: transcription start site
PPMB: peaks per megabase
MYC: MYC proto-oncogene
BRCA1: BRCA1, DNA repair associated
CCND1: cyclin D1
KRAS: KRAS proto-oncogene
SMAD4: SMAD family member 4
PRDM1: PR/SET domain 1
NKX2-1: NK2 homeobox 1
TP63: tumor protein p63
FOXC1: forkhead box C1
